# The clinical relevance of different antiphospholipid antibody profiles in pediatric rheumatology patients

**DOI:** 10.1186/s12969-024-00954-8

**Published:** 2024-04-26

**Authors:** Jheel Pandya, Karen Onel, Doruk Erkan

**Affiliations:** 1 Department of Pediatric Rheumatology, Hospital for Special Surgery, 535 E 70th Street, 10021 New York, NY USA; 2 Hospital for Special Surgery, Barbara Volcker Center for Women and Rheumatic Disease, Weill Cornell Medicine, 535 E70th Street, 10021 New York, NY USA

**Keywords:** Pediatrics, Rheumatology, Antiphospholipid syndrome, Thrombosis

## Abstract

**Background:**

The clinical relevance of different antiphospholipid antibody (aPL) profiles, including low level anticardiolipin (aCL) and anti-β_2_-glycoprotein-I (aβ_2_GPI) antibodies, is ill-defined in the pediatric population. Our purpose is to describe the demographic, clinical, and laboratory characteristics of aPL positive pediatric patients based on different aPL profiles.

**Findings:**

In this single center retrospective cohort study, based on the screening of our pediatric (age ≤ 18) rheumatology electronic medical records (2016–2022), we identified patients who had at least one “positive” aPL (lupus anticoagulant [LA], aCL IgG/M, or aβ_2_GPI IgG/M) result. Patients were grouped into high- (LA positive and/or aCL/aβ_2_GPI IgG/M > 40U [ELISA]) and low-risk (LA negative and aCL/aβ_2_GPI IgG/M 20-39U) aPL profiles; those with persistently positive aPL were descriptively analyzed for demographic and clinical characteristics. Of 57 included patients, 34 (59%) had initial high- and 23 (40%) had initial low-risk profiles. Based on subsequent aPL results available in 42/57 (74%) patients, 25/27 (93%) in the high-, and 7/15 (47%) in the low-risk groups remained still positive. Of these 32 patients with persistently positive aPL, moderate-to-large vessel or microvascular thrombosis occurred in nine (28%) patients with high-risk and in none with low-risk aPL profiles; non-thrombotic aPL-related manifestations were reported in 15 (47%) patients with persistent aPL positivity.

**Conclusion:**

An initial high-risk aPL profile was persistent in approximately 90% of our cohort, a third of whom had thrombosis, and half had non-thrombotic aPL manifestations. Our results underscore the need for a large-scale effort to better characterize aPL-related manifestations in pediatric patients with persistent high-risk aPL-profiles.

**Supplementary Information:**

The online version contains supplementary material available at 10.1186/s12969-024-00954-8.

## Background

Antiphospholipid syndrome (APS) is a systemic autoimmune disorder occurring due to antibodies against phospholipid-binding plasma proteins (antiphospholipid antibodies [aPL]), mainly lupus anticoagulant test (LA), anticardiolipin antibodies (aCL), and anti-β_2_-glycoprotein-I antibodies (aβ_2_GPI).

Traditionally, the diagnosis and classification criteria for APS have been focused on moderate-to-large vessel thrombosis and obstetric complications. However, growing evidence suggests that aPL can also be associated with a wide range of microvascular and non-thrombotic manifestations [1–3]. Antiphospholipid antibody positive patients may also develop skin manifestations (e.g., livedo reticularis/racemosa and cutaneous ulcers), renal disease (aPL-nephropathy), neurologic involvement (chorea), cardiac valve abnormalities, and hematologic abnormalities (thrombocytopenia and hemolytic anemia) [2]. Several pediatric studies have highlighted the importance of early recognition of these microvascular and non-thrombotic manifestations, as they may more commonly be the initial presentation, when compared to adults [4]. These microvascular and non-thrombotic manifestations can significantly impact the quality of life and overall health outcomes of affected pediatric patients [5].

Given the limited knowledge regarding aPL profiles and clinical phenotypes in pediatric aPL-positive patients, the aim of this study was to describe the demographic and clinical characteristics (including microvascular and non-thrombotic manifestations) of aPL-positive pediatric patients based on different aPL profiles.

## Findings

### Methods

In this single-center retrospective cohort study, using electronic medical records, we identified aPL “positive” pediatric patients (≤ 18 years) who were seen at our rheumatology clinic between 2016 and 2022. For patient identification, our initial criterion for aPL “positivity” was at least one abnormally flagged aPL result above the normal range. Initially, we considered those patients with only one positive aPL result solely to identify aPL-positive patients within our pediatric rheumatology clinic who may or may not have persistently positive aPL results. Further analyses were based on patients who had persistently positive aPL results. Local or external laboratories were utilized to test for lupus anticoagulant, and a combination of Diluted Russell Viper Venom Time (DRVVT) and activated partial thromboplastin time (aPTT) were used to determine LA positivity. Enzyme-linked immunosorbent assay (ELISA) was utilized for aCL and aβ_2_GPI testing.

First, we assessed the initial and subsequent (when available) aPL results (type, isotype, and level). For the purpose of analysis, based on the initial test results, we categorized patients into high-risk (LA positive and/or aCL/aβ_2_GPI IgG/M ≥ 40U [ELISA]) and low-risk (LA negative and aCL/aβ_2_GPI IgG/M 20-39U) aPL profiles. We systematically collected data on whether positive aPL patients had repeat testing, if repeat testing resulted in persistently positive aPL, and whether their risk profiles remained high or low on repeat testing.

Secondly, we evaluated our persistently aPL-positive cohort (LA positive and/or aCL/aβ_2_GPI IgG/M ≥ 20U twice at least twelve weeks apart) for demographics, aPL-related clinical characteristics including moderate-to-large vessel thrombosis, pre-defined microvascular disease, non-thrombotic aPL-related manifestations, and for concomitant systemic autoimmune diseases. We included skin manifestations (livedo reticularis/racemosa, and cutaneous ulcers), aPL-nephropathy based on most recent definitions [6], hematologic abnormalities (thrombocytopenia defined as platelet count < 150,000/µl twice with no other concurrent explanation, autoimmune hemolytic anemia defined as hemolytic anemia with positive direct Coombs testing), neurologic involvement (migraines, seizures, chorea and cognitive dysfunction), and cardiac valve abnormalities (thickening and vegetations).

Data were analyzed descriptively. Count measures were summarized as frequency and percentages. Continuous measures were summarized as mean and standard deviation.

## Results

Among the initially aPL-positive patients (*n:*113), 56 were excluded due to very low aCL/aβ_2_GPI IgG/M positivity (< 20U). The excluded patients were not further analyzed for clinical events. Of the remaining 57 patients, 34 patients had initial high-risk aPL profiles (with mean follow up of 4.4 +/- 5 years) and 23 had initial low-risk aPL profiles (with mean follow up of 3.2 +/- 3 years) (Table 1). Based on subsequent aPL results available in 42/57 (74%) patients, 25/27 (93%) in the high-, and 7/15 (47%) in the low-risk groups remained still positive.

Thrombosis occurred in 9/32 (32%) patients (four venous, one arterial and venous, one intracardiac, two microvascular, and one superficial venous), all of whom had initially high-risk aPL profiles (eight with positive LA, and five with triple aPL positivity). No patients with thrombosis had initial low-risk aPL profiles (Tables 2 and 3). Patients with thrombosis were mostly in their adolescent years (12–18) at time of event, and five (56%) had associated autoimmune SLE.

Non-thrombotic aPL-related manifestations were reported in 15 (47%) patients with persistent aPL positivity; 14 (93%) of which had high-risk aPL profiles, and LA positivity (Tables 2 and 3). Neurological manifestations were the most common (migraine: 6 patients, chorea: 1), followed by thrombocytopenia (*n*:5), and autoimmune hemolytic anemia (*n*:4), cardiac valve disease (*n*:2), and livedo reticularis/racemosa (*n*:2).

## Discussion

Our study demonstrated that an initial high-risk profile is persistent in more than 90% of our cohort, while an initial low-risk profile was less likely to be repeated at our institution, and also less likely to remain positive when repeated. Of those with persistent aPL, one-third had associated thrombosis, all of which were associated with initial high-risk profiles. Furthermore, nearly half of the patients with persistently positive aPL also had microvascular and/or non-thrombotic manifestations, the majority of which were in patients with LA positivity.

Given the transient aPL-positivity during infections, persistent (at least 12 weeks apart) positivity is required for APS classification [7], a concept also important for APS diagnosis. Based on our clinical experience, supported by the literature, low level aCL/aβ_2_GPI positivity is less likely to be persistent when repeated [7]. Similarly, we found that approximately half our patients with low (20-39U) aCL/aβ_2_GPI levels had negative results when repeated.

Patients with high-risk aPL profiles, defined as persistent LA positivity and/or aCL/aβ_2_GPI IgG/M levels ≥ 40U, are at higher risk for thrombosis, compared to those with low risk aPL profiles (negative LA and aCL/aβ_2_GPI levels 20-39U) [8, 9]. In our study, approximately one-fourth of pediatric patients with high-risk aPL profiles had a history of thrombosis, consistent with previous studies in adult APS populations, highlighting the importance of high-risk aPL profiles as a marker of thrombotic risk [8, 9].

Nearly half of our pediatric patients with persistent aPL positivity exhibited non-thrombotic aPL-manifestations. These findings align with emerging evidence suggesting that aPL can contribute to a broader range of clinical manifestations beyond thrombosis in children [1, 10], just as has been identified in the adult population. Some studies even suggest that non-thrombotic neurological manifestations such as migraines and chorea may be seen in higher frequencies in children with aPL positivity [11–14]. Therefore, although controversial in the adult APS literature, these neurological non-thrombotic manifestations are included in our study as they have been reported in the pediatric aPL population [1, 10, 15, 16]. Recognizing and characterizing these non-thrombotic manifestations in pediatric APS is crucial for appropriate classification, management and improved quality of life for affected patients.

Our study is not without limitations, which should be recognized. Firstly, the retrospective nature of the study introduces inherent biases and limitations associated with data collection and analysis. Although different laboratories (local or external) were utilized to test for LA; a combination of DRVVT and PTT-LA were used in all patients to determine LA positivity. Also due to the retrospective nature of the study, information on indications for performing aPL testing, especially if performed at an outside or referring institution, was unavailable. The patients seen in our pediatric rheumatology clinic were tested either during or after evaluation for a connective tissue disease. It is also important to recognize that we may not see all patients with isolated hematologic or neurologic manifestations of aPL in our pediatric rheumatology clinic. Furthermore, all aPL profiles were reported in our data according to the first positive result as testing practices vary among physicians. Due to varying aPL testing patterns and insidious presentation of many non-thrombotic manifestations, the exact temporal relationship between aPL results and these manifestations was difficult to capture. Therefore, non-thrombotic manifestations were captured if they were present at any time during follow up of these patients. In aPL-positive patients with lupus, it can be difficult to accurately estimate if thrombotic and non-thrombotic aPL manifestations are attributable to aPL, SLE, or both; given the descriptive nature of the study we decided to report all manifestations independent of the underlying lupus classification. Additionally, our study was conducted at a single center, which may limit the generalizability of the findings to other populations. Future multicenter, prospective studies involving larger cohorts are warranted to validate our results and provide a more comprehensive understanding of aPL profiles in pediatric APS.

## Conclusions

In conclusion, our study contributes to the growing body of literature on the clinical relevance of different aPL profiles in pediatric rheumatology patients, shedding light on the persistence of high-risk aPL profile positivity, subsequent testing patterns, and the occurrence of thrombotic, microvascular, and non-thrombotic aPL-related manifestations. Our findings highlight the importance of further research and a collaborative international effort to better characterize aPL-related manifestations, define pediatric-specific classification criteria, and optimize management strategies for aPL-positive pediatric patients.

**Table 1:** Follow-up antiphospholipid antibody (aPL) results in 57 patients with at least one high- or low-risk aPL profile

	Initial LA + and/or aCL/aβ_2_GPI ≥ 40U	Initial LA- and aCL/aβ_2_GPI 20-39U
	(*n*:34)	(*n*:23)
Mean Follow up (+/- SD) (y)	4.4 ± 5.0	3.2 ± 2.9
Subsequent aPL positive / # of patients with repeat aPL	25/27 (93%)	7/15 (47%)
• Repeat LA positive / Initial LA positive	18/27 (67%)	2/0^#^
• Repeat aCL/aβ_2_GPI positive / Initial aCL/aβ_2_GPI positive	19/23 (83%)	5/15 (36%)*

LA: lupus anticoagulant; aCL: anticardiolipin antibody; and aβ_2_GPI: anti-β_2_ glycoprotein-I antibody. 93% (25) of patients in the high-risk profile group continued to have positive results on repeat testing, with 18 (67%) of 27 initial LA positive patients remaining LA positive, and 19 (83%) of 23 initial aCL and/or aβ_2_GPI positive patients remaining positive for aCL and/or aβ_2_GPI. On the other hand, 47% (7) of patients in the low-risk group continued to have positive results on repeat testing, 5 (36%) of which continued with positive aCL and/or aβ_2_GPI. ^#^Two patients who initially only had low-titer positive aCL (one with IgM and one with IgG), later developed positive LA. *Two patients had subsequent high-risk aPL titers with aCL ≥ 40U

**Table 2:** Demographic, clinical, and laboratory characteristics of 32 patients with persistent antiphospholipid antibody (aPL) profiles

	Triple aPL Positive	LA Positive with/without aCL or aβ_2_GPI	LA Negative with aCL and/or aβ_2_GPI
	(*n*:9)	(*n*:12)	(*n*:11)
**aCL/aβ**_**2**_ **GPI Level**			
aCL/aβ_2_GPI IgG/M 20-39U	1 (11%)	3 (25%)	7 (64%)
aCL/aβ_2_GPI IgG/M ≥ 40U	8 (89%)	2 (17%)	4 (36%)
**Demographics**			
Mean Age at Presentation	13.2 ± 4.65	14.7 ± 2.57	13.8 ± 4.95
Female	7 (78%)	11 (92%)	10 (91%)
White	5 (56%)	7 (58%)	6 (55%)
Black	2 (22%)	-	1 (9%)
Hispanic	1 (11%)	3 (25%)	2 (18%)
Asian	1 (11%)	1 (8%)	1 (9%)
**Lupus Classification**	5 (56%)	7 (58%)	5 (45%)
**Thrombosis**	5 (56%)	3 (25%)	1 (9%)*
Venous	4	-	1
Arterial	-	-	-
Both Venous and Arterial	-	1	-
Microvascular	1	2	-
**Non-thrombotic aPL Manifestations** ^$^	5 (56%)	9 (75%)	1 (9%)**
Thrombocytopenia^#^	1	4	-
Autoimmune Hemolytic Anemia	2	2	1
Cardiac Valve Disease	-	1	-
Livedo Reticularis/Racemosa	1	1	-
Migraines***	3	2	-
Chorea ***	1	-	-

LA: lupus anticoagulant; aCL: anticardiolipin antibody; and aβ_2_GPI: anti-β_2_ glycoprotein-I antibody. *In a patient with aCL IgM > 40U initially, then 20-30U on repeat testing; ^#^Platelets < 150,000 /µl twice with no other diagnosis. **Autoimmune hemolytic anemia occurred in a patient with initially low-risk profile, who later developed a high-risk profile with aCL IgM > 40. ***Controversial aPL-related manifestations, which may be more relevant in pediatric population. ^$^Of 15 patients with non-thrombotic aPL manifestations, nine (60%) had lupus classification (six with cytopenia) and six (40%) did not have an SLE classification (four with cytopenia)

**Table 3:** Demographic and clinical characteristics of nine persistently antiphospholipid antibody (aPL) positive patients with moderate-to-large vessel and/or microvascular thrombosis

Age^#^	Sex	Associated Autoimmune Disease	Thrombotic Manifestations	APS Related Non-thrombotic Manifestations*	aPL Profile^**^
**12**	F	N/A	Intrahepatic IVC thrombus, PE, popliteal artery thrombosis	Thrombocytopenia, AIHA, livedo racemosa	LA
**12**	F	SLE	aPL-nephropathy^$^	AIHA, livedo reticularis, migraine	LA aCL IgG 20-39U & IgM ≥ 40U aβ2GPI IgM ≥ 40U
**14**	F	SLE-Like Disease	DVT (x2), PE	N/A	LA aCL IgG ≥ 40U aβ2GPI IgG ≥ 40U
**16**	F	N/A	DVT/PE	N/A	LA aCL IgM ≥ 40U aβ2GPI IgM ≥ 40U
**16**	F	N/A	DVT (x2)	N/A	aCL IgM ≥ 40U
**17**	F	SLE	Intracardiac thrombus	Cardiac valve disease	LA
**17**	M	N/A	Superficial vein thrombosis	Migraine	LA aCL IgM ≥ 40U aβ2GPI IgG ≥ 40U
**18**	F	SLE	DVT	Migraine	LA aCL IgG ≥ 40U aβ2GPI IgM ≥ 40U
**18**	F	SLE	Livedoid vasculopathy related skin ulcer	Thrombocytopenia	LA

F: Female; M: Male; DVT: Deep venous thrombosis; PE: Pulmonary embolism; IVC: inferior vena cava; SLE: systemic lupus erythematosus; AIHA: autoimmune hemolytic anemia. LA: lupus anticoagulant; aCL: anticardiolipin antibody; and aβ_2_GPI: anti-β_2_ glycoprotein-I antibody. All patients had abnormal aPL testing within one year of first thrombotic event. After initial event, five patients were on aspirin, six patients were on low molecular weight heparin (LMWH) (two of whom were later placed on Warfarin), and six of the patients were on hydroxychloroquine (only one of whom did not have SLE or SLE-like disease). ^#^Patients were all post-pubertal or in their late adolescent years; however, Tanner staging was not available on documentation. *Non-thrombotic manifestations were included if they were not attributable to another diagnosis; **aPL profile at time of first event. ^$^The aPL-nephropathy was established based on renal biopsy

### Electronic supplementary material

Below is the link to the electronic supplementary material.

Supplementary Material 1

## Data Availability

The datasets used and/or analyzed during the current study are available from the corresponding author on reasonable request.

## Abbreviations

APS: Antiphospholipid Syndrome
aPL: Antiphospholipid antibody
aCL: Anticardiolipin
aβ_2_GPI: Anti-β_2_-glycoprotein-I
LA: Lupus Anticoagulant
DVT: Deep venous thrombosis
PE: Pulmonary embolism
IVC: Inferior vena cava
SLE: Systemic lupus erythematosus
AIHA: Autoimmune hemolytic anemia
DRVVT: Diluted Russell Viper Venom Time
aPTT: activated partial thromboplastin time
