# Supplementary material for: The clinical relevance of different antiphospholipid antibody profiles in pediatric rheumatology patients
Source: Pediatr Rheumatol Online J. 2024 Apr 26;22:46. doi: 10.1186/s12969-024-00954-8 (PMC11046946; doi:10.1186/s12969-024-00954-8)
Supplement: Supplementary file 1 — Supplementary Material 1 [file 12969_2024_954_MOESM1_ESM.docx]

**Response to Reviewers**

**Reviewer #1:**

**Suggestions.
Methods. Please explain the reason researchers considered only de presence of LA or high titers of aCL or aB2GPI to be classified as high risk and no other clinical or serological features. Also, considering just one result, instead of two separate from some weeks, is a problem, because it does not allow to avoid false positive results.**

- We agree it is very important to stratify patients based on their risk of thrombus. We also agree that clinical features are also important while determining the risk of (recurrent) thrombosis in aPL-positive patients.
- We utilized the laboratory parameters to classify patients into high and low risk categories, based on laboratory profiles, only as a start point in our analysis. We considered those patients with only one positive result initially only to identify patients within our pediatric rheumatology clinic, and to determine what our clinic practices have been doing in repeating this testing. We only included patients who had at least two positive results in further analyses (as seen in Tables 2 and 3), to avoid false positive patients. We clarified this point in the methods (Page 4) with the following statement: “*Initially, we considered those patients with only one positive aPL result solely to identify aPL-positive patients within our pediatric rheumatology clinic who may or may not have persistently positive aPL results. Further analyses were based on patients who had persistently positive aPL results.”*

**Reviewer #2:**

**This is a concise, well-written and informative report of pediatric patients with aPL positivity from a single center who were subjected to a retrospective analysis. As stated by the authors, the aim of this study was to describe the demographic and clinical characteristics (including microvascular and non-thrombotic manifestations) of aPL-positive pediatric patients based on different aPL profiles.**

**The strength of this study as well as other reports of small cohorts of pediatric APS patients is that they become available for analysis by the International committees working to develop both classification and diagnostic criteria for this disease in children. This and the need for larger scale prospective studies has been clearly stated in the text.**

**This is an important area in pediatric APS that needs more attention and more data in order to establish criteria and recommendations for diagnosis and treatment. Additional information should be provided by the authors in order to potentiate this effort and provide the most information for international consensus committees to achieve these goals (the more detailed information provided even on a small cohort, the more accurate the conclusions when large scale analysis via meta-analytic studies takes place).**

- We appreciate the positive feedback of the reviewer and we agree with all the points above.

**It is imperative that the information in this report appear in the most useful format for these future endeavors. The text and tables discuss the demographics of this cohort but only address the age and sex of the patients. One of the reasons that multicenter and international efforts are necessary is the ability to represent diverse genetic backgrounds. In order for this report to be useful to those efforts, the authors should provide the race/ethnicity of the patients in their cohort. Data on ethnicity and race is lacking but is probably easily inserted on the text and/or tables. It is not likely that a population from NYC is homogeneous and thus this information again would be useful to any further analysis by other groups in combining and matching datasets.**

- We want to thank the reviewer for this extremely important point. We have added additional race/ethnicity information in Table 2.

**Out of interest, did any of the 56 excluded patients with low titer aPL have thrombosis or non-thrombotic events? I assume they were all LA negative. What was the age range of these patients?**

- The 56 excluded patients were not further analyzed for clinical events due to the nature of our exclusion criteria. All of these patients were, by definition, LA negative, as they would have been included in the cohort if they were positive for LA. Unfortunately, we did not analyze the age range of these patients as they were excluded from further analysis.

**The authors do not define the tests used to determine the Lupus anticoagulant such as DRVVT, Hexagonal-Phase, PTT-LA as well as the cutoff values established by the authors. This is very important as some of these assays are influenced by factors associated with inflammatory conditions such as factor 8 levels. Some LA assays may be positive in F8 deficiency and false negative in patients with endothelial cell activation (i.e. in active SLE) with elevated F8 levels. Please provide information on the LA assays used as each is specific for difference aspects of the coagulation cascade. Were there age-related cutoffs for LA assays (does the lab use pediatric controls?) This is important due to developmental hemostasis. While post-pubertal patients could utilize the adult normal range values this may not be appropriate for pre-pubertal patients (as levels of many coagulation factors in pre-pubertal patients are similar to those in patients on warfarin).**

- We agree that this is a very important point as various methods are used to determine LA positivity and results can be influenced by other factors. Because different laboratories (local or external) were utilized to test for lupus anticoagulant, we did not have a standardized LA testing method in our retrospective study. However, a combination of DRVVT and PTT-LA were used in all our patients to determine LA positivity. We added this point to the limitation section of the discussion on Page 6 to include: “*Although different laboratories (local or external) were utilized to test for lupus anticoagulant; a combination of DRVVT and PTT-LA were used in all patients to determine LA positivity. Also due to the retrospective nature of the study, information on indications for performing aPL testing, especially if performed at an outside or referring institution, was unavailable.”*

**The last paragraph of the Results section discusses the non-thrombotic aPL-related manifestations in this cohort. The 1st paragraph of the discussion emphasizes the predominance of LA positivity in these patients with non-thrombotic manifestations. Was this associated with one LA assay type vs another?**

- Please see our response to the previous question.

**Page 5; Line 11: change (Table 2,3) to ( Tables 2 and 3) as indicated in the following paragraph on that page.**

- Thank you, this change has been made.

**Page 5; Line 13-14: “Patients with thrombosis were mostly in their adolescent years (12-18) at time of event,”. Given this age range, most likely these patients were post-pubertal, but were there any pre-pubertal patients with thrombosis? This is important in regards to development hemostasis and risk for thrombosis and should be mentioned briefly.**

- The ages of patients with thrombosis are shown in Table 3. Of these patients, the two youngest patients had events at age 12, and both were post-pubertal at time of presentation to our clinic. All other patients were in their late adolescent years at presentation. We added this information to Table 3 as a footnote to state: “^#^*Patients were all post-pubertal or in their late adolescent years; however, Tanner staging was not available on documentation.”*

**Table 1: in the last footnote “* Two had subsequent high-risk…” would sound better as “* Two patients had subsequent high-risk…”**

- Thank you, this change has been made.

**Table 2: under demographics, this might be a good place to add ethnicity and race information as well as pre-pubertal vs post-pubertal status.**

- Thank you, race and ethnicity has been added to Table 2. Pubertal status for all the patients was not added due to missing Tanner stage documentation in our pediatric rheumatology clinic. We added to Table 3 as a footnote to state: “^#^*Patients were all post-pubertal or in their late adolescent years; however, Tanner staging was not available on documentation.”*

**Table 3: instead of a hyphen for missing data, the authors could probably state “none” or “N/A”**

- Thank you, this change has been made.

**This is another place where race and ethnicity could be added as an additional column.**

**Unless there is a good reason for the order in which these patients are presented in this Table, the authors could group that patients either by age (ascending or descending) or by SLE or not SLE**

- Thank you for the suggestion, we have re-ordered the patients based on age in Table 3.

**In the footnote the authors state “F: Female” and although it is obvious, please include “M: Male”**

- Thank you, this change has been made.

**References:**

**Perhaps a more recent reference for #12 would be helpful which also addresses the topic of development hemostasis:**

**Rumsey DG, Myones B, Massicotte P. Diagnosis and treatment of antiphospholipid syndrome in childhood: A review. Blood Cells Mol Dis. 2017 Sep;67:34-40. doi: 10.1016/j.bcmd.2017.02.009. Epub 2017 Feb 28. PMID: 28283251.**

- Thank you so much for this suggestion, this reference has been added.

**The authors need to check the accuracy of the citations, for example reference # 3 was not published in “1926” it was published in 2020. And reference #12 does not have a date listed at all (published in 2015). Please make sure all the references are in the same format.**

- Thank you, the references have been updated.

**Reviewer #3:**

**1. What were initial indications to perform aPL testing in patients seen in the rheumatology clinic? Did all patients present with aPL related clinical manifestations or was aPL testing performed as a part of the routine screening in patients with connective tissue disease.**

- Thank you for this important question. Given the retrospective nature of the study, unfortunately we could not systematically collect the information about indications to perform aPL testing. We added this information to the limitation section of the discussion on Page 6 to include: “*Also due to the retrospective nature of the study, information on indications for performing aPL testing, especially if performed at an outside or referring institution, was unavailable.* *The patients seen in our pediatric rheumatology clinic were tested either during or after evaluation for a connective tissue disease.”*

**2. The patients' population was biased as the study included only patients seen in the rheumatology clinic. The authors should describe the referral process and specify if the study excluded patients who initially presented with neurological, hematological or cardiac manifestations.**

- This is a very important consideration, however, as discussed above, unfortunately we do not have this information. The limitations section of the discussion has been updated to incorporate this point. On Page 6 and 7 we added: “*Also due to the retrospective nature of the study, information on indications for performing aPL testing, especially if performed at an outside or referring institution, was unavailable. The patients seen in our pediatric rheumatology clinic were tested either during or after evaluation for a connective tissue disease. It is also important to recognize that we may not see all patients with isolated hematologic or neurologic manifestations of aPL in our pediatric rheumatology clinic*.”

**3. The authors should provide the time points of thrombotic events. I wonder how many patients had thrombosis at the time of initial aPL determination and how many during the follow-up. It would be important to provide also data on SLE disease activity and underlying therapy at the time of thrombotic events.**

- Table 3 describes patients with medium to large vessel thrombosis and microvascular disease along with the aPL profile at the time of first event. Based on the available data, all patients had abnormal aPL testing within one year of first thrombotic event, we included this as a footnote under Table 3 to state: “*All patients had abnormal aPL testing within one year of first thrombotic event*.”
- Because repeat testing and patterns of testing for aPL vary greatly among physicians, it was difficult to record similar data for non-thrombotic manifestations. Therefore, all aPL profiles were reported in our data according to the first positive result.
- Due to the retrospective nature of this study, there was no systematic recording of the SLE disease activity. However, based on reviewer’s request, we have added a footnote to Table 3 stating: “*After initial event, five patients were on aspirin, six patients were on low molecular weight heparin (LMWH) (two of whom were later placed on Warfarin), and six of the patients were on hydroxychloroquine (only one of whom did not have SLE or SLE-like disease).”*

**4. Were non-thrombotic aPL-related manifestations more common in patients with underlying** **SLE?**

- This is an excellent question. To clarify this point, we added a footnote to Table 2 stating: *“^$^Of 15 patients with non-thrombotic aPL manifestations, nine (60%) had lupus classification (six with cytopenia) and six (40%) did not have an SLE classification (four with cytopenia).”*

**5. Please describe in more detail the methodology used for aPL testing or provide appropriate reference.**

- Thank you, additional information on aPL testing was added to our methods section on page 4 to include: *“Local or external laboratories were utilized to test for lupus anticoagulant, and a combination of DRVVT and PTT-LA were used to determine LA positivity. Enzyme-linked immunosorbent assay (ELISA) was utilized for aCL and aβ_2_GPI testing.*”

**Reviewer #4:**

**This is a descriptive, single-center study of aPL profiles and associated clinical characteristics in a convenience sample of children evaluated within a pediatric rheumatology clinic. There is relatively sparse data regarding aPL positivity and progression to APS in the pediatric population, and therefore this report adds incremental knowledge and highlights the importance of evaluating non-thrombotic manifestations. However, the study design limits the conclusions that can be made and does not provide estimations of risk. The report would also benefit from methodological clarifications and reference checks.**

- We appreciate the positive feedback of the reviewer.

**Methods**

**The “high-risk” and “low-risk” designations are potentially confusing since they differ slightly from other standard definitions (no article is referenced in the Methods). For “high-risk”, it is clear that a single LAC+ with or without aCL/b2GP Ig meets the authors’ definition, but does the use of “and/or” imply that any single aCL/B2gp Ig >40 is also sufficient to meet the authors’ definition (i.e. not double positivity)? If so, some additional justification could be provided.**

- Thank you for this important point. Based on the 2023 ACR/EULAR APS classification criteria, “moderate” level aCL/aβ_2_GP1 positivity is defined as 40-79 ELISA, which is the initial level we used to define these patients as high-risk for an event. We initially only identified patients who had at least one positive aPL result in order to avoid missing any patients who had subsequent aPL testing done outside our institution as well as to determine the pattern of practice for repeat testing in these patients at our institution. In further analysis, we only included patients with persistent positivity, in order to be consistent with current literature and classification criteria for APS. We clarified these points on Page 4 as: *“Initially, we considered those patients with only one positive aPL result solely to identify aPL-positive patients within our pediatric rheumatology clinic who may or may not have persistently positive aPL results. Further analyses were based on patients who had persistently positive aPL results.”*

**Page 5, lines 55-59; The authors’ definition of high-risk profiles is referenced here as being associated with higher risk for thrombosis in the literature, but the cited references do not appear to use the same definitions of exposure.**

- Thank you, update to these references has been made. Now we included the following reference: *Barbhaiya M, Zuily S, Naden R, et al. 2023 ACR/EULAR Antiphospholipid Syndrome Classification Criteria. Arthritis and Rheumatology. Published online 2023. doi:10.1002/art.42624*

**It’s not clear whether clinical manifestations were evaluated over a certain period of time before or after which initial aPL positivity was identified – similarly, for those with persistent positivity, when were the clinical outcomes evaluated in relation to when they met the definition for persistence? In the table 3 footnote it states that for those with thrombotic events, aPL profiles were evaluated at the time of first event, but how was this determined for those with non-thrombotic manifestions?**

- Because patterns of testing for aPL vary greatly among physicians, retrospective nature of the study, and the fact that non-thrombotic manifestations can often be insidious, it was difficult to determine the exact temporal relationship between aPL and non-thrombotic manifestations. However, all patients analyzed had persistent aPL-positivity and these manifestations were included in the analysis if they were present at any time during the follow up.
- We recognize this as a limitation of the study, and have included this discussion in the limitations section on Page 7 to include: *“Due to varying aPL testing patterns and insidious presentation of many non-thrombotic manifestations, the exact temporal relationship between aPL results and these manifestations was difficult to capture. Therefore, non-thrombotic manifestations were captured if they were present at any time during follow up of these patients.”*

**Results**

**Page 4, lines 58-59. Do the 27 patients that “fit the high-risk profile group” refer to 27/42 patients who had repeat aPL results that were persistently “high-risk” profiles, or does it mean that 27 of the 42 patients with repeat testing belonged to the initial high-risk group?**

- We agree this is an important clarification. We have changed the wording of this statement to *“27 of the 42 patients with repeat testing belonged to the initial high-risk group”.*

**Table 2 – The table title references persistent high or low risk profiles, but the table columns are stratified in a different way, which adds to the ambiguity mentioned above about the definition of high risk.**

- Yes, we recognize this table implies a different definition of high- and low-risk profile, and have adjusted the title accordingly. Thank you for the suggestion.

**Table 2 – were the non-thrombotic aPL manifestations also at the time of initial aPL testing? Or did these occur over some specified period of follow-up after initial testing?**

- On chart review, these manifestations were recorded if they were present at any time during the follow up of these patients. We included in the limitation section of the discussion on Page 7 as: *“Due to varying aPL testing patterns and insidious presentation of many non-thrombotic manifestations, the exact temporal relationship between aPL results and these manifestations was difficult to capture. Therefore, non-thrombotic manifestations were captured if they were present at any time during follow up of these patients.”*

**Table 3 – Since this is a descriptive study, additional detail could be included in this table that would be helpful for interpretation. For example, what other medications were they exposed to at the time (especially those that could potentially also affect aPL titers)?**

- This is a very important point. Per the reviewer’s suggestions, we have added a footnote to Table 3 stating: “*After initial event, five patients were on aspirin, six patients were on low molecular weight heparin (LMWH) (two of whom were later placed on Warfarin), and six of the patients were on hydroxychloroquine (only one of whom did not have SLE or SLE-like disease).”*

**Table 3 - Please specify whether it was suspected vs. established (pathologic confirmation) aPL-nephropathy, and if suspected, how lupus nephritis was excluded as an equally or more likely diagnosis. Similarly, how did the authors adjudicate whether cytopenias in SLE patients were not due to SLE disease activity?**

- Thank you for this suggestion. We have added a footnote to Table 3 to include: *“The aPL-nephropathy was established based on renal biopsy.”*
- We agree with the reviewer that cytopenias can be difficult to attribute to lupus or aPL in an aPL-positive lupus patient. Given the descriptive nature of our study and relatively low number of patients (and those with cytopenias), we now included the number of patients with lupus and cytopenias (Table 2) stating: *“^$^Of 15 patients with non-thrombotic aPL manifestations, nine (60%) had lupus classification (six with cytopenia) and six (40%) did not have an SLE classification (four with cytopenia).”*
- We have also added the following statement to our limitations on Page 7: “*In aPL-positive patients with lupus, it can be difficult to accurately estimate if thrombotic and non-thrombotic aPL manifestations are attributable to aPL, SLE, or both; given the descriptive nature of the study we decided to report all manifestations independent of the underlying lupus classification.”*

**Discussion**

**There are some nice reviews included in the references, however some key references from the primary literature appear to be missing, including the actual published results of the Ped-APS registry 10.1542/peds.2008-1209**

- Thank you, we have updated the references accordingly.

**Reviewer #5:**

**This is a well-written and concise report of a large single-center experience with 57 children who had positive anti-phospholipid antibodies. As has a been seen in adults, presence of a lupus anticoagulant and/or high titer antibody to beta-2-glycoprotein 1 and and/or cardio Lipkin (> 40 U/l) was present in all who had thrombotic disease, or micro vascular manifestations of the syndrome, and in those with persistent positivity. clinical features of their population were discussed.**

**The diagnosis of childhood APS is an important medical question that remains incompletely answered, but is highly important as the risks of thrombotic disease is devastating, but anti ovulation, the treatment for this condition, Carrie's the risk of hemorrhage, particularly in active children and teens. This manuscript is limited (as the authors discuss) by retrospective data analysis and limited sample size, however, for a rare disease, it contributes important information to the literature.**

- Thank you very much, we agree with these statements. We hope to contribute to the limited, yet growing knowledge of this rare disease in the pediatric population.


  ********
